# A Multi-Element-Doped Porous Bioactive Glass Coating for Implant Applications

**DOI:** 10.3390/ma14040961

**Published:** 2021-02-18

**Authors:** Christie Y. K. Lung, Mohamed M. Abdalla, Chun Hung Chu, Iris Yin, Sofiya-Roksolana Got, Jukka P. Matinlinna

**Affiliations:** Faculty of Dentistry, The University of Hong Kong, Hong Kong 999077, China; mohamabd@hku.hk (M.M.A.); chchu@hku.hk (C.H.C.); risxyin@hku.hk (I.Y.); sofiyagot@gmail.com (S.-R.G.); jpmat@hku.hk (J.P.M.)

**Keywords:** titanium, bioactive glass, implant coating, antibacterial, cell cytotoxicity

## Abstract

Objectives: The objectives of the study were (1) to develop a novel multi-element-doped porous 58S bioactive glass coating for titanium implants and (2) to investigate the physiochemical, cell cytotoxic and antibacterial properties of this novel coating for titanium implants. Methods: This study employed the sol–gel method to develop a silver-, cobalt (II) oxide- and titanium dioxide-doped 58S bioactive glass coating. The surface topography and in vitro bioactivity of the new bioactive glass-coated implants were studied using scanning electron microscopy (SEM) and energy-dispersive X-ray spectroscopy. The surface nanohardness and coating degradation were evaluated using atomic force microscopy (AFM) and inductively coupled plasma atomic emission spectroscopy (ICP-AES), respectively. The cell cytotoxicity was assessed using cell viability of osteoblast-like mouse cells. The antibacterial property was examined using colony-forming units (CFUs) of the implant coating against *Porphyromonas gingivalis*. Results: The multi-element-doped porous 58S bioactive glass-coated titanium implant was synthesized. SEM showed that calcium phosphate was formed on the novel coating but not on the 58S bioactive glass coating. The mean surface nanohardness of the novel coating and the 58S coating were 124 ± 24 and 50 ± 17 MPa, respectively (*p* < 0.001). ICP-AES showed that the releases of Si, Ca and P ions of the novel coating were significantly higher than that of a 58S bioactive glass-coated implant. No significant difference in cell cytotoxicity was found between the novel coating and the 58S coating (*p* > 0.1). The mean CFUs of the novel coating and the conventional coating were 120 × 10^6^ and 49 × 10^6^ /mL. Conclusion: A novel multielement-doped porous bioactive glass coating for titanium implants was developed. The coating displays promising biocompatibility and antibacterial activity. Clinical significance: the coating can be used to improve the clinical success of dental implants for patient care if it shows success in clinical trials.

## 1. Introduction

Titanium is widely used for various dental and orthopedic implants because of its excellent biocompatibility, inertness and biomechanical properties. However, the main drawbacks of titanium implants are slow bone regeneration and instability during the healing stage of implantation (secondary stabilization) [1,2]. In clinical practice, the conventional surface treatments of titanium implants are grit-blasting, acid etching and grit-blasting plus acid etching. These surface treatment methods increase the surface roughness, which enhances the contact area between the implant and the bone tissues. Therefore, these methods improve osseointegration compared to machined-only titanium implants [3].

Osseointegration is an important factor that has significant influence on the success of long-term implant stability. However, another factor, which is the major concern in dental and orthopedic implantology, has negative influence on implant stability; this is peri-implantitis. It is an inflammatory disease arising from bacterial biofilm formation on the implant surface. The final outcome could ultimately be implant failure if there is no surgical treatment in time [4]. Surface modification with bioactive materials might enhance bone regeneration and reduce healing time. However, in some cases, such as grit-blasting of a titanium surface, it may also increase the bacterial adhesion [5]. Bioactive glass is able to form a chemical bond with the bone tissue [6]. Currently, the main focus is to improve its mechanical properties, as it is brittle and has low fracture toughness [7]. Further improvements include the antibacterial and biological activity [7].

Bioactive glass 58S has been applied for bone tissue engineering because of its good bioactivity, biodegradability and biocompatibility without adverse effects. However, the major drawbacks of 58S are its brittleness, non-antibacterial activity and low fracture toughness [8]. Substitution of other elements for silicon in the bioactive glass can improve the physicochemical and biological properties.

Cobalt has been reported to activate hypoxia inducible factor-1 (HIF-1), which can improve the vascularization and therefore enhance osteogenesis, i.e., bone regeneration [9,10]. Doping with titanium has been reported to enhance cell proliferation and differentiation [11]. Certain metal or metal oxide nanoparticles, especially silver, are very effective against a broad spectrum of bacteria [12].

Porous materials have been applied in nanotechnology as they exhibit well-defined structural and morphological features, have various surface chemical functionalities and have a very high surface area [13]. The porous structure can facilitate bone tissue ingrowth and enhance osseointegration [14]. There are several techniques to generate porous structure: one of them is the use of organic polymers as the porous structure-directing agents. After the porous phase formation, the polymer is removed by calcination [13].

In this study, the effect of porous structure and doping with silver, cobalt (II) oxide and titanium dioxide on the physicochemical and biological properties of a 58S bioactive glass coating were evaluated. The porous bioactive glass coatings were prepared by the sol–gel method. The surface nanohardness and in vitro degradation of the coatings were evaluated. The in vitro mineral calcium phosphate formation, cell cytotoxicity and antibacterial properties were also examined. The null hypotheses tested were 1) the nanohardness of the bioactive glass coatings would not differ between test groups, 2) the cell cytotoxicity of the coatings would not be affected by the doped elements and 3) the antibacterial properties of the coatings would not be affected by the doped elements.

## 2. Materials and Methods

### 2.1. Materials

Titanium discs (c.p. grade 2, Sigma Aldrich, St. Louis, MO, USA), tetraethyl ortho-silicate (Sigma Aldrich), calcium nitrate tetrahydrate (Sigma Aldrich), triethyl phosphate (Sigma Aldrich), silver nitrate (AR grade, Accu-Chem, Melrose Park, IL, USA), cobalt (II) nitrate (Sigma Aldrich), titanium (IV) iso-propoxide (98%, Strem Chemicals, Newburyport, MA, USA) and pluronic polymer F127 (Sigma Aldrich) were used to prepare the bioactive glass-coated titanium samples.

### 2.2. Surface Pretreatment of Titanium Discs

Titanium discs with a diameter of 25.4 mm and thickness of 5.1 mm were polished with silicon carbide papers using a polishing machine (Lunn Major). The samples were rinsed ultrasonically in 70% ethanol and dried. The samples were heated to 60 °C in 5 M sodium hydroxide solution for 24 h. Next, the samples were heated to 600 °C for 1 h after the treatment [15].

### 2.3. Preparation of 58S and Doped Porous Bioactive Glass Coatings

The porous bioactive glass was prepared according to the modified method reported previously [16]. Tetraethyl ortho-silicate, calcium nitrate tetrahydrate, triethyl phosphate, silver nitrate, cobalt (II) nitrate, titanium (IV) iso-propoxide and 0.5 g of pluronic polymer F127, a porous structure-directing agent, were used. The reagents were added into 10 mL absolute ethanol according to different glass compositions (Table 1). Then, 1.0 mL of 0.5 M nitric acid was added. The reaction mixture was stirred at 40 °C for 6 h. The bioactive glass coatings on titanium samples were applied by spin coating at a spin rate of 3000 rpm for 30 s. Two consecutive depositions (100 µL × 2) were performed. The samples were then thermally treated at 700 °C for 3 h to remove the polymer.

### 2.4. Characterization of the Bioactive Glass Coatings

The surface topography of bioactive glass coatings was examined by SEM (SU1510, Hitachi, Tokyo, Japan) with an operational voltage of 15 kV. The surface elemental composition was analyzed by EDX (SDD3310, IXRF, Austin, TX, USA).

### 2.5. Surface Nanohardness Test

The nanohardness tests of the bioactive glass coatings were carried out with an AFM system (Dimension Edge, Bruker, Billerica, MA, USA) modified from a previous study [17]. A maximum load of 55.3 µN was applied with a loading rate of 0.5 µm/s to obtain an indentation mark. Five nanoindentations were performed randomly, at different positions for each coating. Thereafter, the mean nanohardness value was calculated.

### 2.6. In Vitro Coating Degradation Test

The samples were immersed in 50 mL of 50 mM Tris–HCl buffer (pH 7.3) solution in polyethylene tubes and incubated at 37 °C, as modified from a previous method [18]. After immersing for 1, 7 and 14 d, the concentrations of Si, Ca and P ions were analyzed in duplicates for each time period using inductively coupled plasma atomic emission spectroscopy (Spectro Arcos, Spectro, Boschstr, Kleve, Germany). The mean concentrations of Si, Ca and P ions were calculated.

### 2.7. In Vitro Bioactivity Test

The samples were immersed in 35 mL of Hank’s balanced salt solution (HBSS) (Gibco, Carlsbad, CA, USA) in polyethylene containers and incubated at 37 °C for 7, 14 and 21 days [19]. The approximate HBSS solution used is given by the formula SA/V = 0.4 cm^−1^, where SA = disc surface area and V = volume of HBSS [20]. After immersion in HBSS, the samples were dried, rinsed with deionized water and air-dried. Mineral calcium phosphate formation was examined by SEM and EDX.

### 2.8. Cell Cytotoxicity Assay

The cell cytotoxicity was assessed using the indirect method [21]. The MC3T3-E1 osteoblast-like mouse cells (Tissue Culture Laboratory, HKU Faculty of Dentistry) were cultured in α-minimal essential medium (α-MEM, Gibco) supplemented with 10% fetal bovine serum (Gibco), streptomycin (50 g/mL) and a 1% antibiotic mixture (300 mg/mL streptomycin and 5 mg/mL amphotericin B). The incubation took place at 37 °C in humidified 5% CO_2_. Three discs from each group were placed into a 6-well plate filled with 5 mL of α-MEM according to ISO standard 10993-5 [22]. Samples were incubated at 37 °C in humidified 5% CO_2_ for 72 h. The extracts released were collected. Two concentrations, 100% and 50% dilutions, were filtered through a sterile 0.22 µm syringe filter (Acrodisc, Pall). The MC3T3-E1 cells (4 × 10^4^ cells/mL) were seeded in 96-well plates and incubated for 24 h. Next, the medium was removed, and 100 µL of each material extract was added to the seeded cells and incubated for 24 h.

The cell cytotoxicity was evaluated by the MTT assay (Sigma-Aldrich). The MTT stock solution was prepared by adding 2 mL PBS to 10 mg MTT. After removal of the extracts, 10 μL of MTT stock and 100 μL of α-MEM were added to each well. Three wells without seeded cells served as the background. The cells were incubated at 37 °C for 4 h. After that, 85 μL of medium was removed and then 50 μL of dimethyl sulfoxide was added to each well, followed by incubation at 37 °C for 10 min. The absorbance at 540 nm was registered with a spectrophotometer (SpectraMax M2, Molecular Devices, San Jose, CA, USA) and the tests were carried out in duplicate.

### 2.9. Antibacterial Test

Antibacterial activities were evaluated using the plate-counting method [23]. *Porphyromonas gingivalis* ATCC 33277 was used. Bacteria were incubated in a fluid nutrient medium at 37 °C for 24 h. Next, 3 mL of bacterial suspension (10^6^ CFU/mL) was seeded on each disc (two discs per group). The plates were sealed and incubated anaerobically for 5 days in an anaerobic chamber (Forma Anaerobic System, Thermo Scientific, Waltham, MA, USA). After that, the inoculum was removed and serial dilutions of *P. gingivalis* suspension were obtained from each well. Then, 50 µL from each dilution was inoculated on Colombia blood agar plates supplemented with vitamin K and hemin and cultured anaerobically for 5 days. The whole procedure was done in duplicate. The number of colonies formed on the agar plates was counted. The colony-forming unit (CFU/mL) of each disc was calculated using the following formula: (number of colonies counted per plate × dilution factor)/volume of culture plate.

### 2.10. Statistical Analysis

Data were coded and entered using the statistical package for the Social Sciences (SPSS) version 26 (IBM, Armonk, NY, USA). Data was summarized using the mean and standard deviation. Comparisons between groups were done using analysis of variance (ANOVA) with the multiple comparisons post hoc test [24]. The p-values less than 0.05 were considered as statistically significant.

## 3. Results

### 3.1. Bioactive Glass Coating Characterization

**Figure 1:**
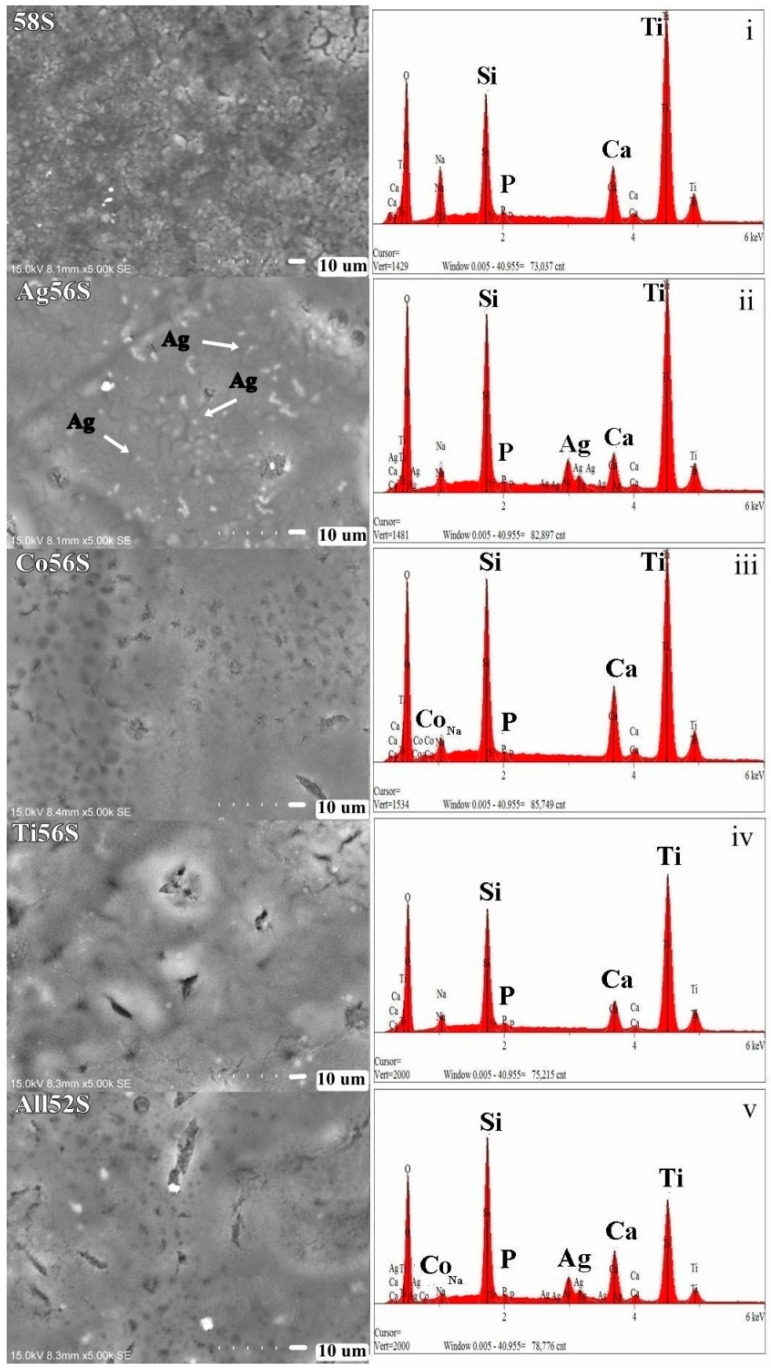
SEM micrographs (5000×) and EDX spectra of different bioactive glass coatings: i. 58S, II. Ag56S, iii. Co5S, iv. Ti56S and v. All52S. The arrows indicate the “Ag” particles.

The surface topography of all the bioactive glass coatings is shown in Figure 1. A uniform deposition of bioactive glass particles on the 58S bioactive glass coating was found. For doped porous bioactive glass coatings, a smooth surface morphology with a porous structure could be observed. Some micro cracks were observed for the porous bioactive glass coatings. As shown by the EDX spectra (Figure 1), the chemical composition for each bioactive glass coating was comprised of Si, Ca, P, Ag, Co and Ti, respectively.

### 3.2. Nanohardness of the Bioactive Glass Coatings

The nanohardness of the coatings measured is reported in Table 2. The lowest surface hardness was measured to be 49.99 ± 17.37 MPa on the 58S coating. The highest surface hardness was measured to be 139.66 ± 49.52 MPa on the Ag56S coating. The one-way ANOVA analysis showed that the mean nanohardness values of Ag56S (*p* < 0.001), Co56S (*p* < 0.01), Ti56S (*p* < 0.005) and All52S (*p* < 0.01) were all significantly higher than that of the 58S coating.

### 3.3. Degradation Test

**Figure 2:**
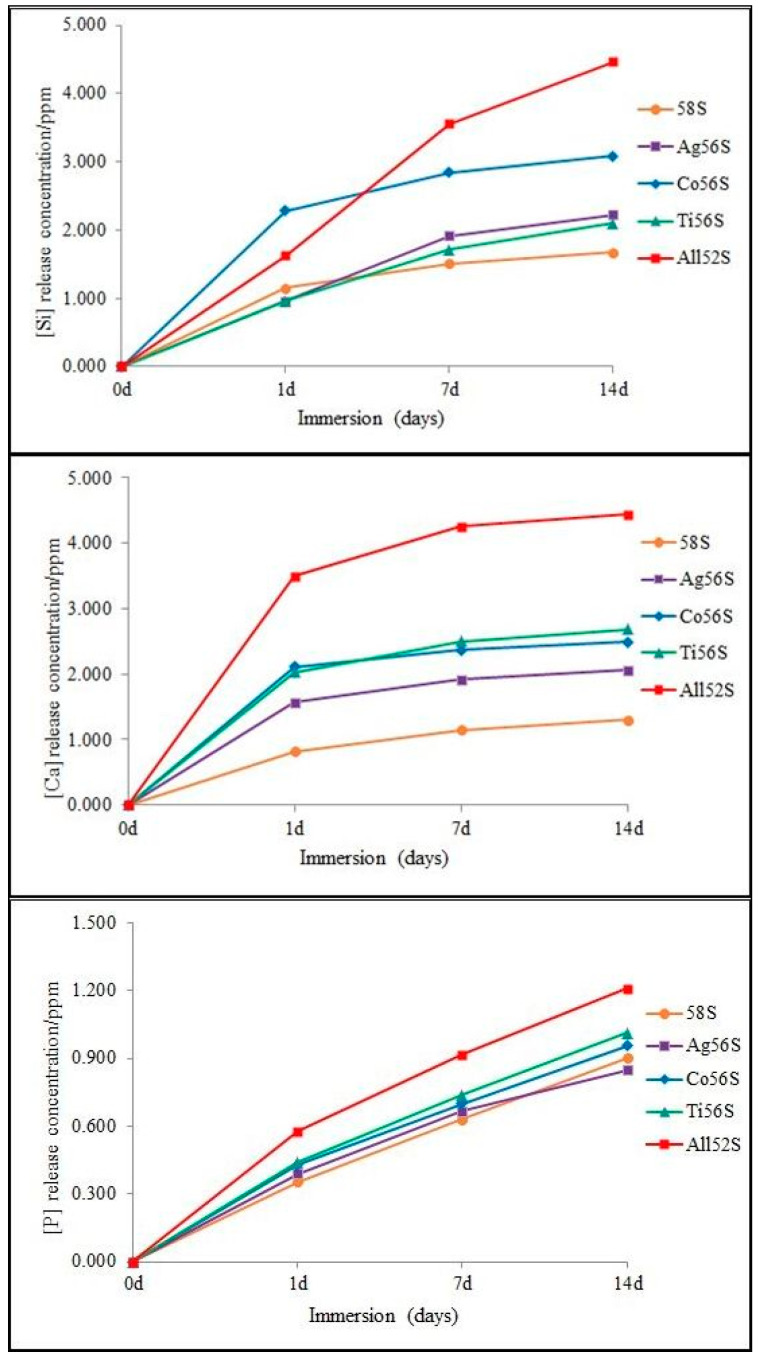
The Si, Ca and P ion release of the bioactive glass coatings after 1 d, 7 d, and 14 d of immersion in Tris–HCl solution.

The release of Si, Ca and P ions from the coatings are shown in Figure 2. All coatings displayed different increasing rates of Si, Ca and P ion release. The degradation of porous bioactive glass coatings, except P released from the Ag56S coating, was faster than that of the 58S bioactive glass coating at 14 d of immersion in Tris–HCl solution. The highest release rate of Si, Ca and P ions was observed for the All52S bioactive glass coating at 14 d.

### 3.4. In Vitro Bioactivity Test

**Figure 3:**
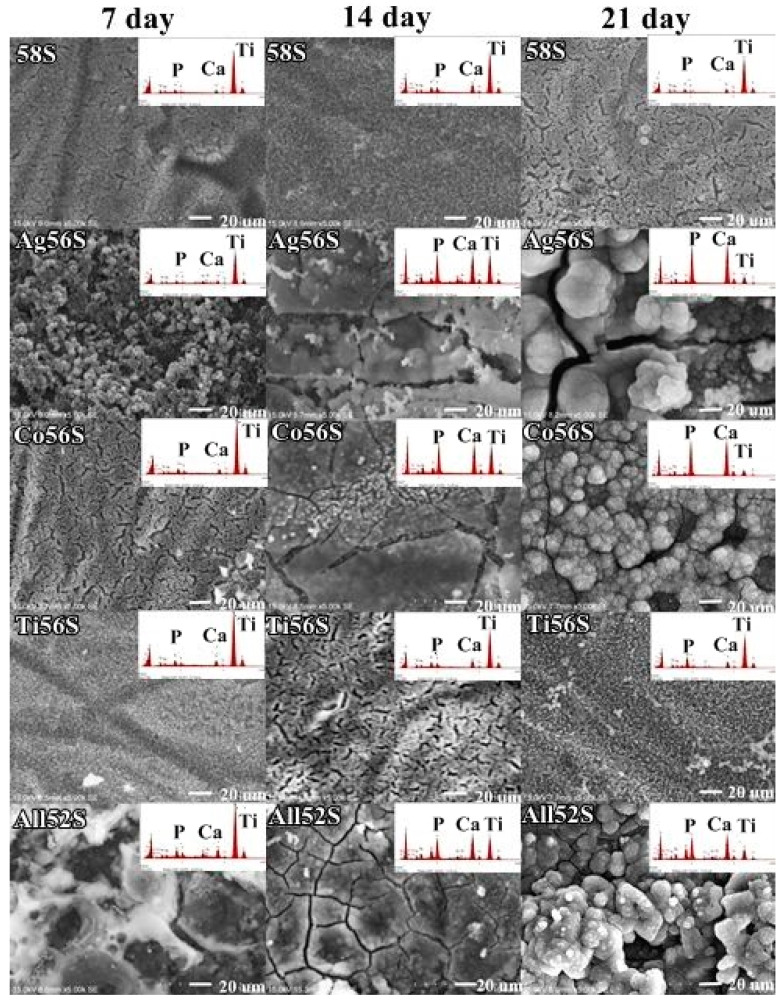
SEM micrographs (5000×) of the bioactive glass coatings immersed in HBSS at 7, 14 and 21 d. Mineral formation with Ca and P can be observed for the Ag56, Co56 and All52S coatings.

Figure 3 shows the SEM images of the mineral calcium phosphate formation on different bioactive glass coatings after immersion in HBSS. There was no apatite formation at 7, 14 or 21 d of immersion for 58S coating and the Ti56S coating. For the Co56S coating, there was no mineral calcium phosphate formation after 7 d of immersion. For the Ag56S and All52S coatings, a very small part of the surface was covered by mineral calcium phosphate particles at 7 d of immersion. More mineral calcium phosphate was formed at 14 and 21 d of immersion for the Ag56S, Co56S and All52S coatings. The EDX spectra (Figure 3, insert figure) shows the Ca and P peaks of the mineral calcium phosphate particles formed on various bioactive glass coatings with respect to the time of immersion. The intensity of Ca and P peaks increases as more mineral calcium phosphate particles are deposited on the Ag56S, Co56S and All52S coatings.

### 3.5. Cell Cytotoxicity Test

**Figure 4:**
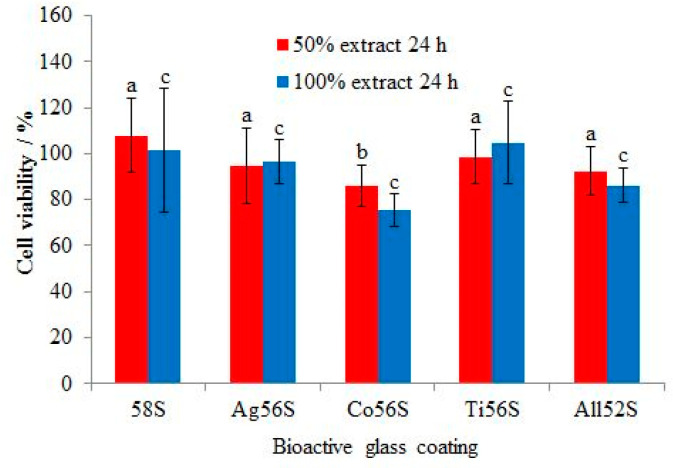
Cytotoxicity of bioactive glass coatings on MC3T3-E1 osteoblast-like mouse cells. Different letters mean a significant difference between groups (*p* < 0.05).

Figure 4 shows the percentage of viable MC3T3-E1 mouse cells that had grown on the bioactive glass-coated Ti surface. The lowest cell growth was observed for the Co56S coating at 24 h of culture for 50% and 100% extract concentrations. The highest cell growth was observed for the 58S coating at 24 h of culture for the 50% extract concentration and for the Ti56S coating for the 100% extract concentration. A high cell viability (>70%) was found for all groups. The cell growth on the 58S coating was not significantly higher than on Ag56S (*p* > 0.1), Ti56S (*p* > 0.5) and All52S (*p* > 0.1) at 24 h for the 50% extract concentration. The cell growth on the 58S coating was significantly higher than Co56S (*p* < 0.05) at 24 h for the 50% extract concentration. The cell growth on the 58S coating was not significantly higher than Ag56S (*p* > 0.9), Ti56S (*p* > 0.9) Co56S (*p* > 0.05) and All52S (*p* > 0.5) at 24 h for the 100% extract concentration.

### 3.6. Antibacterial Test

**Figure 5:**
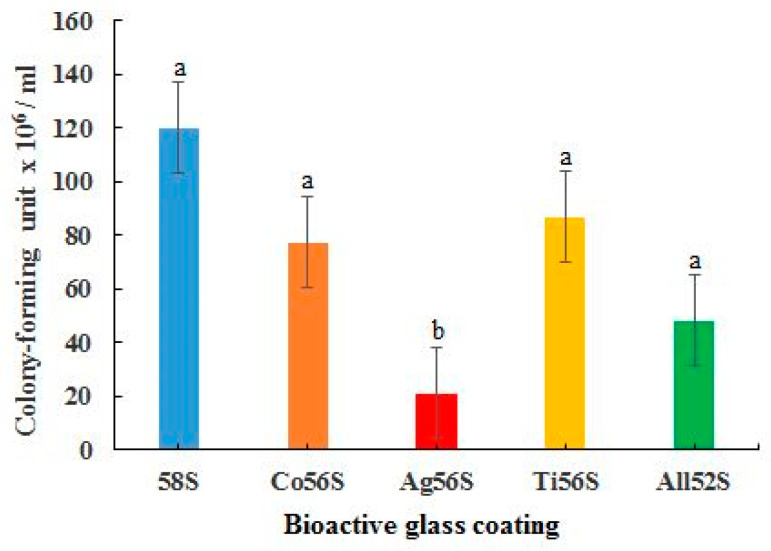
Antibacterial activity against *Porphyromonas gingivalis* for the bioactive glass coatings. Different letters mean the groups are significantly different (*p* < 0.05).

In Figure 5, the Ag56S coating was observed to be most effective in inhibiting the growth of *Porphyromonas gingivalis*. The All52S coating was the second most effective in inhibiting the growth of the bacteria. The Ag56S, Co56S, Ti56S and All52S coatings all displayed higher antibacterial activities than the 58S coating. The antibacterial activities of the Co56S (*p* > 0.3), Ti56S (*p* > 0.4) and All52S (*p* > 0.2) coatings were not significantly higher than the 58S bioactive glass coating. The antibacterial activity of Ag56S (*p* < 0.05) coating was significantly higher than that of the 58S coating.

## 4. Discussion

The current study reported on a multi-element-doped porous bioactive glass coating that combines the (1) antibacterial properties of nano Ag, (2) improvement of vascularization of the bone tissue to enhance osteogenesis (Co) and (3) enhancement of the cell proliferation and differentiation (Ti). Substitution of these elements affects the physicochemical and biological properties of bioactive glass [25].

The surface pretreatment of Ti discs with NaOH followed by thermal treatment generated a bioactive titanate layer, Na_2_Ti_6_O_13_ [15]. An in vivo study showed that the bioactive titanate layer improved bone bonding to the implant [26]. After the bioactive glass is coated, a bi-layer structure is formed. Once the bioactive glass layer is integrated with the bone tissue, the bone tissue connects to the inner bioactive titanate layer and forms a strong bond with the implant.

For titanium and titanium metal alloy (e.g., Ti–6Al–4V) prosthesis, a bioactive glass coating on it can form a protective layer [27]. This minimizes the corrosion from oral fluid and the release of metal ions into the oral cavity. This may lead to allergic reactions or a cytotoxic effect [28].

The difference between the coefficient of thermal expansion (CTE) of titanium (~9.0–9.6 × 10^−6^ °C^−1^) and bioactive glass (~13–17 × 10^−6^ °C^−1^) is expected to induce residual tensile stress in the coating. This may affect the bonding adhesion. Approaches to decrease the CTE of bioactive glass include increasing the silica content and partially replacing it with other oxides, such as MgO or TiO_2_ [29]. Therefore, 58S bioactive glass was studied instead of 45S5 bioactive glass, and SiO_2_ was partially replaced by TiO_2_. So, the CTE of the multi-element-doped bioactive glass was decreased (it was not measured in this study). This minimizes the difference between the CTE of titanium and this multi-element-doped bioactive glass and the negative effect on the coating adhesion.

The surface roughness of the bioactive glass coatings depends on the composition of the bioactive glass; method of preparation, e.g., melting vs sol–gel method; and coating deposition methods, e.g., plasma spraying vs spin coating. Surface roughness could affect the bone–implant interactions. A rough surface is beneficial for biomechanical anchorage in bone tissue to improve implant stability [29]. The mean surface roughness, Ra, for bioactive glass and silicate-based coatings on titanium metal and alloys are in the range of 0.4–11.9 µm [30]. The bonding strength of bioactive glass coatings on titanium also depends on the composition of bioactive glass, method of preparation and coating deposition methods. The bonding strength between the bioactive glass coating and titanium is important. The detachment of the coating during implant healing results in slow osseointegration and implant instability. The bonding strengths measured for a bioactive silicate-based coating on titanium alloys are in the range of 17.4–49.8 MPa, with mean values higher than 15 MPa (the minimum bonding strength recommended by the ISO) [30].

The surface hardness of all the doped porous bioactive glass coatings was improved. The first null hypothesis was rejected. The Ag, CoO and TiO_2_ nanoparticles could act as nanofiller particles to enhance the surface hardness [31]. Furthermore, TiO_2_ can form a network structure with silica, SiO_2_ (i.e., –O–Si–O–Ti–O–) with a replacement of the lighter Si by the heavier Ti. This appears to increase the density of the bioactive glass; moreover, the glass structure becomes more compact, i.e., is decreased in volume. As a result, the surface hardness of the TiO_2_-doped bioactive glass is increased [32]. There was no significant improvement of surface nanohardness for the All52S bioactive glass coating compared with the other three single-element-doped bioactive glass coatings. Probably, the decrease in silica content (from 56% to 52%) was more significant than doping of the three elements in affecting the surface hardness.

The degradation of bioactive glass releases ions with respect to the time period of immersion in Tris–HCl buffer solution. The degradation rate of the 58S coating was slower than for all the doped porous bioactive glass coatings. This may be due to the increase in the mole percentage of SiO_2_ in 58S bioactive glass. This would increase the network bridging oxygen and mean more energy is required to break the network [33]. The other reason may be due to the porous structure and thus the increased surface area promoting glass dissolution and ion release [34].

The in vitro formation of mineral calcium phosphate by immersion in simulated body fluid (SBF) such as Hank’s balanced salt solution is very useful to evaluate the biomineralization activity of the materials. It is a good prediction of the in vivo bone-forming ability of the bioactive materials [35]. During the immersion in SBF, the surface silica network is broken down to form hydrated silicates (–Si–OH). The calcium phosphate nucleates on the surface –Si–OH groups [36]. The Ag56S, Co56S and All52S coatings improved the in vitro apatite formation as compared to the 58S coating. There was no apatite formation on the 58S coating, even after 21 d. This could be due to the higher content of SiO_2_ in 58S bioactive glass. Higher content of SiO_2_ decreases the dissolution rate of the glass to form hydrated silicates [33]. On the other hand, the doped elements would also affect the apatite nucleation through the competitive cation exchange with Ca^2+^ on the bioactive glass surface [37].

The cell cytotoxicity was assessed using an indirect method. The particles released from the coatings interact with the cells. It was noted that the CoO-doped porous bioactive glass coating released Co^2+^ ions that were more cytotoxic towards MC3T3-E1 mouse osteoblast-like cells than the other coatings. In another study, Co^2+^ ions induced oxidative stress and affected the morphology and cell viability of MG63 cells [38]. The generation of intracellular reactive oxygen species (ROS) can cause oxidative damage to the cells [39]. The Ag- and TiO_2_-doped porous bioactive glass coatings exhibited lower cell cytotoxicity than the CoO-doped porous bioactive glass coating. The cell cytotoxicity of the bioactive glass coatings depends on the doped element. The second null hypothesis was rejected. The cell viability of the All52S porous bioactive glass coating was higher than 80% for both extract concentrations; this means the coating displayed a good biocompatibility.

The antibacterial performance of the bioactive glass coatings was affected by the doped elements. The third null hypothesis was rejected. Both the CoO- and TiO_2_-doped porous bioactive glass coatings exhibited some antibacterial activities towards *P. gingivalis*. The Ag-doped porous bioactive glass coating was the most effective in inhibiting the bacteria, followed by the All52S bioactive glass coating.

It is noteworthy that the underlying antibacterial mechanisms of silver and metal oxides are different. The antibacterial mechanisms of silver nanoparticles are based on the direct contact of silver nanoparticles and the release of silver ions from silver nanoparticles [40]. The amount of silver ions released from silver nanoparticles depends on the surface area of the nanoparticles. The released silver ions interact with the cell membrane, which causes the disruption of the cell membrane. The silver ions bind to the ribosomes and cause their denaturing, thus inhibiting protein synthesis. The silver ions deactivate the respiratory enzyme on the cytoplasmic membrane, and thus adenosine triphosphate (ATP) production is terminated. The cell membrane is denatured by the reactive oxygen species, which are produced when silver ions bind to the cell membrane. Silver ions and reactive oxygen species bind to deoxyribonucleic acid and interfere with cell replication and multiplication. Silver nanoparticles can accumulate in the cell wall and cause membrane denaturation. They can dephosphorylate tyrosine residues on the peptide substrates. This disrupts signal transduction and leads to cell apoptosis.

As for the metal oxide nanoparticles, the antibacterial mechanisms are attributed to the following [41]: (1) Cell membrane disruption by electrostatic interactions: the positive charge of the metal cation in metal oxide nanoparticles is attracted to the negative charge carried on the surface of the bacterial cell membrane. The binding of the membrane polymers with cationic metal oxide nanoparticles leads to toxicity to the bacteria. (2) metal/metal ion homeostasis is disturbed. When excess metal ions bind to the bacteria, this causes a disorder in metabolic functions. (3) When metal oxide nanoparticles enter bacteria, they generate reactive oxygen species. ROS mainly inhibit respiratory proteins. They damage iron–sulfur (Fe–S) clusters in adenosine triphosphate (ATP), which suppresses ATP production. On the other hand, the metal ions released from metal oxide nanoparticles oxidize glutathione, which protects bacteria from oxidative stress. The process generates more ROS. The imbalance between oxidants (ROS) and antioxidants (glutathione) causes oxidative stress in the bacteria and inhibits bacterial activity. (4) The metal ions can catalyze the oxidation of amino acid side chain functional groups in proteins. This process causes oxidative protein damage and leads to loss of enzyme activity. (5) Metal oxide nanoparticles interact with nucleic acids, such as genomic and plasmid DNA. They can disrupt the replication process and suppress the cell division of microbes. They can also affect the signal transduction in bacteria such that the phosphotyrosine is dephosphorylated. This results in the obstruction of bacterial growth.

After the insertion of a titanium implant, there are two competitive processes occurring: bone tissue integration and bacterial adhesion followed by a biofilm formation. The biofilm formation on the implant surface hinders the bone tissue cell functions [42]. Furthermore, biofilm formation would also lead to implant failures. An ideal antibacterial and bioactive implant coating is expected to inhibit the bacterial activity effectively while being nontoxic to the bone tissue cells and promoting cell growth for bone tissue regeneration and accelerated healing, i.e., secondary implant stabilization [43]. If the antibacterial agents are cytotoxic to tissue cells, it will cause cell death and slow down the cell proliferation for tissue regeneration. Therefore, a balance between the antibacterial activity and the cell cytotoxicity is essential for the design of the antibacterial implant coating.

The limitations of the present study are as follows: (1) The multi-element-doped bioactive glass coating showed good in vitro biocompatibility and antibacterial activity. Further in vivo study would be needed to prove the performance in animals. (2) Only *Porphyromonas gingivalis* was tested in the antibacterial test. Other bacterial species related to periodontitis, such as *Tannerella forsythia*, *Prevotella intermedia*, and *Staphilococcus aureus*, should be tested in future studies.

## 5. Conclusions

A novel biocompatible multi-element-doped porous bioactive glass with low cytotoxicity was developed. This multi-element-doped and porous bioactive glass coating displays improved physicochemical and biological properties compared to a 58S bioactive glass coating. The novel bioactive glass has the potential to be used as a dental implant coating material for titanium.

## Figures and Tables

**Table 1:** Compositions of each bioactive glass prepared (by mole percentage; mol%).

Bioactive Glass Formulation	Chemical Composition/mol%				
	SiO_2_	CaO	P_2_O_5_	TiO_2_	CoO
58S	58	37	5	0	0
Ag56S	56	37	5	0	0
Co56S	56	37	5	0	2
Ti56S	56	37	5	2	0
All52S	52	37	5	2	2

**Table 2:** Nanohardness of the bioactive glass coatings.

Bioactive Glass Group	Mean Nanohardness ± SD/MPa
58S	49.99 ± 17.37 ^a^
Ag56S	118.71 ± 19.1 ^b^
Co56S	107.18 ± 8.29 ^b^
Ti56S	139.66 ± 49.52 ^b^
All52S	124.44 ± 24.7 ^b^

a, b: Different letters mean the groups are significantly different (*p* < 0.05).

## Data Availability

The data presented in the study are available on request from the corresponding author.

## Abbreviations

MTT: 3-(4:5-dimethylthiazol-2-yl)-2,5-diphenyltetrazolium bromide

58S: 58%SiO_2_:37%CaO:5%P_2_O_5_ bioactive glass

Ag56S: 56%SiO_2_:37%CaO:5%P_2_O_5_:2%Ag silver-doped bioactive glass

Co56S: 56%SiO_2_:37%CaO:5%P_2_O_5_:2%Co cobalt oxide-doped bioactive glass

Ti56S: 56%SiO_2_:37%CaO:5%P_2_O_5_:2%Ti titanium dioxide-doped bioactive glass

All52S: 52%SiO_2_:37%CaO:5%P_2_O_5_:2%Ag:2%Co:2%Ti silver-, cobalt oxide- and titanium dioxide-doped bioactive glass
